# Systemic metastasis in malignant solitary fibrous tumor of the liver: two case reports and literature review

**DOI:** 10.3389/fonc.2024.1418547

**Published:** 2024-10-02

**Authors:** Pengcheng Wei, Chen Lo, Jie Gao, Jiye Zhu, Xin Sun, Zhao Li

**Affiliations:** ^1^ Department of Hepatobiliary Surgery, Peking University People’s Hospital, Beijing, China; ^2^ Beijing Key Surgical Basic Research Laboratory of Liver Cirrhosis and Liver Cancer, Peking University People’s Hospital, Beijing, China; ^3^ Peking University Center of Liver Cancer Diagnosis and Treatment, Peking University People’s Hospital, Beijing, China; ^4^ Peking University Institute of Organ Transplantation, Peking University People’s Hospital, Beijing, China; ^5^ Musculoskeletal Tumor Center and Beijing Key Laboratory of Musculoskeletal Tumor, Peking University People’s Hospital, Beijing, China

**Keywords:** solitary fibrous tumor, liver tumor, mesenchymal neoplasms, malignancy, metastasis

## Abstract

Solitary fibrous tumor of the liver (SFTL) is an exceptionally rare mesenchymal tumor, with only 117 cases reported in the literature. While most SFTs are benign, some exhibit malignant behavior, including local recurrence and metastasis. This report presents two cases of SFTL with systemic metastases, both involving prior intracranial tumors. The first case, a 52-year-old woman, discovered a liver mass incidentally during a routine physical exam. Subsequent investigations revealed potential bone metastasis, and biopsy confirmed SFT. She received two TACE procedures, anlotinib targeted therapy, and radiotherapy for the iliac bone lesion, resulting in stable disease with reduction in lesion size. The second case, a 46-year-old man, presented with multiple liver, pelvic, and lung lesions following pelvic tumor resection, with pathology confirming SFT. He was treated with long-term anlotinib therapy, CyberKnife for hepatic, lung, and pelvic lesions, and radiofrequency ablation for hepatic lesions. Postoperative recovery was uneventful, with no tumor progression on follow-up. SFTL presents with atypical clinical and imaging features, and diagnosis requires pathological and genetic confirmation. Radical resection is preferred for solitary tumors, while comprehensive treatment, including surgery and long-term follow-up, is essential for cases with recurrence or metastasis.

## 1 Introduction

Solitary fibrous tumor (SFT), first identified by Klemperer and Rabin in 1931, is a rare tumor originating from fibroblastic mesenchymal tissues, comprising spindle cells and collagen (1). SFT affects a broad age range, including young adults and the elderly, with no significant gender disparity, though some reports indicate a slight female predominance (1, 2). SFT has a relatively low incidence compared to other systemic tumors, typically occurring in serosal-lined organs like the pleura, peritoneum, and retroperitoneum, as well as in non-serosal sites like the central nervous system, orbits, lungs, and mediastinum (3). Solitary fibrous tumor of the liver (SFTL) is exceedingly rare, with only 117 cases documented in medical literature to date. While most SFTLs follow a benign clinical course, they possess malignant potential, with approximately 10-20% categorized as malignant, capable of local recurrence and metastasis (4).

Most SFTL patients exhibit no clear symptoms, though some may have an abdominal mass or mild discomfort, typically found incidentally during exams for other conditions (5). When large, the tumor can pressure nearby liver structures, leading to vague right upper abdominal pain, intermittent dull pain, and right subcostal pressure. SFTL lacks distinctive imaging characteristics, making ultrasound, CT, and MRI non-specific for diagnosing it. Differential diagnosis includes hepatocellular carcinoma, focal nodular hyperplasia (FNH), and vascular ectodermal cell neoplasm, among others. A definitive diagnosis relies on distinct histopathological and immunohistochemical features. Treatment for SFTL varies with the tumor’s characteristics, size, and the patient’s general health. For localized lesions, surgical resection is preferred (6). Additionally, radiotherapy, chemotherapy, interventional therapy, ablation therapy, and targeted immunotherapy are effective treatment options.

However, in-depth research on the clinical diagnosis and treatment of SFTL is lacking. This article summarizes and analyzes the clinicopathological features and prognosis of two SFTL cases at our center, along with previous literature reports, aiming to offer a foundation and reference for SFTL diagnosis and treatment to guide clinical practice.

## 2 Case presentation

### 2.1 Case 1

A 52-year-old female patient was found to have a hepatic space-occupying lesion during a physical examination two weeks ago and reported mild abdominal discomfort. She had a history of an intracranial tumor, with surgery performed many years ago. Pathology suggested a possible hemangiopericytoma. She had no history of liver disease, no family history of genetic disorders, and no family history of tumors. Physical examination revealed stable vital signs and normal cardiopulmonary function. The abdomen was soft, without tenderness or rebound pain, and the liver and spleen were not palpable below the rib margin. Laboratory tests showed normal results for blood, urine, and fecal routine examinations, as well as normal biochemistry. Hepatitis B and C tests were negative, and tumor markers were within normal limits.

An MRI enhancement scan of the upper abdomen, performed prior to hospital admission, revealed multiple liver parenchymal lesions of varying sizes, displaying long T1 and T2 signals. The lesions exhibited uneven signals and inhomogeneous high intensity on DWI. The largest lesion, measuring approximately 10.4×7.8×10.4 cm, protruded significantly from beneath the liver and showed progressive, inhomogeneous, and marked enhancement. Low-density, non-enhancing necrotic areas were observed in the centers of the larger lesions, while smaller lesions showed more uniform enhancement. FDG PET/CT imaging further indicated multiple foci of increased FDG metabolism in the liver, sacrum, T8 vertebrae, and left ilium, suggesting multiple metastatic malignant tumors, with the primary origin possibly in the liver. Some liver foci were poorly demarcated from the inferior vena cava, raising the possibility of tumor thrombus. Scattered solid nodules were also observed in both lungs, raising concerns about potential lung metastasis.

**Figure 1:**
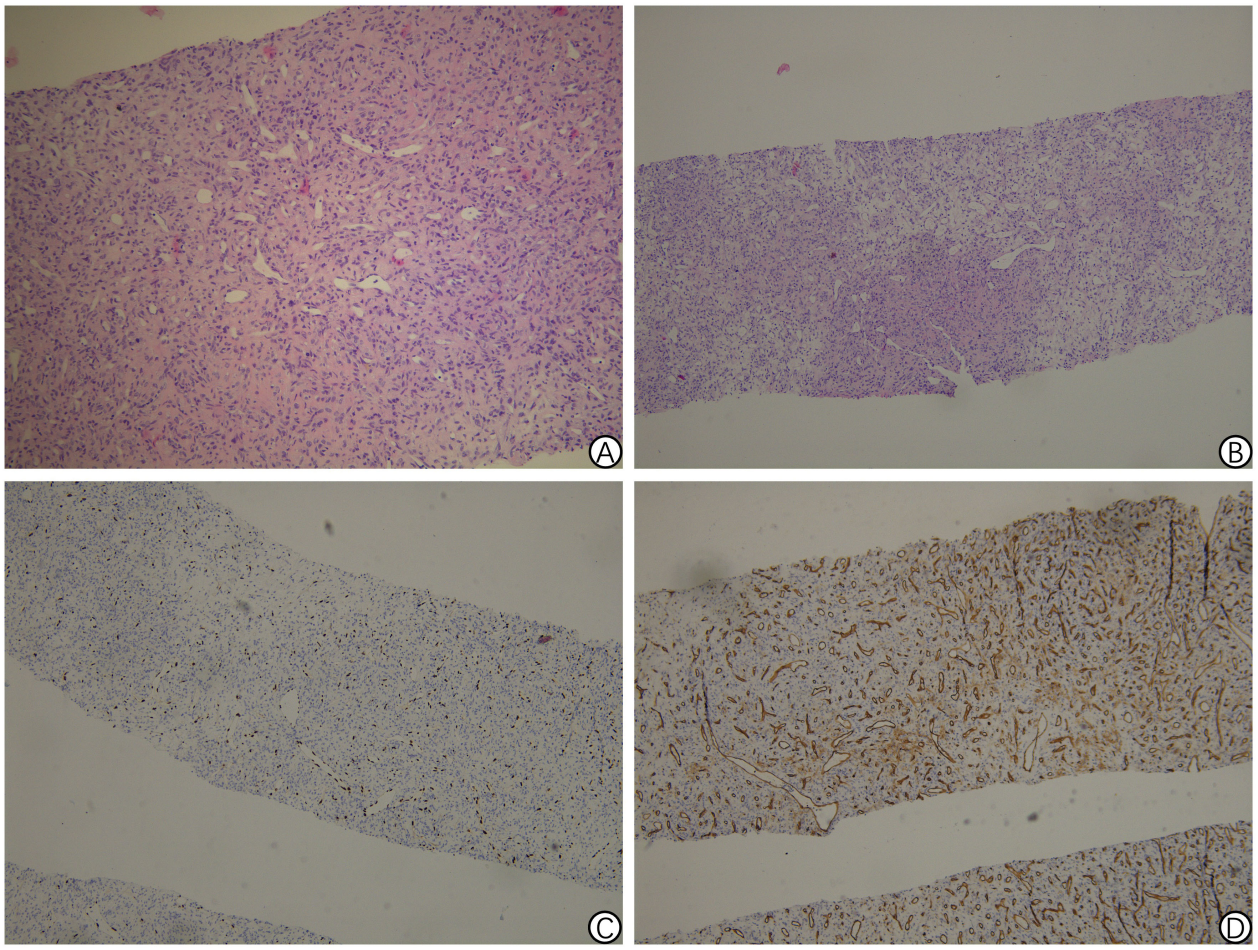
Pathology findings of solitary fibrous tumor of hepatic puncture biopsy in Case 1. **(A)** Proliferation of spindle cells randomly arranged in the abundant stromal collagen (hematoxylin and eosin staining, 200 × magnification); **(B)** Immunohistochemical staining revealing the positive ERG staining in the tumor cells (100 × magnification); **(C)** Immunohistochemical staining showing the positive CD34 staining in the tumor cells (100 × magnification); **(D)** Ki-67 Labeling index of 10%-15% (100 × magnification).

The patient was admitted to the hospital and underwent an ultrasound-guided hepatic biopsy. Pathology revealed tumor invasion in a small liver tissue sample, characterized by spindle-shaped and ovoid tumor cells with mild to moderate atypia. Rare schizonts were observed, along with prominent luminal-like components, an interstitium rich in thin-walled blood vessels, and areas of interstitial hyalinized fibrosis. Immunohistochemistry results were as follows: CK (-), Hepatocyte (-), Arg1 (-), CD31 (+), CD34 (+), ERG (+), CD117 (-), DOG1 (-), PDGFR (-), and Ki-67 (10%+), consistent with a tumor of mesenchymal origin. Refer to **Figure 1**. Further NGS-506 genetic testing identified NAB2-STAT6 fusion, consistent with solitary fibrous tumor (SFT). Refer to **Table 1**.

**Table 1:** The results of the Next-Generation Sequencing (NGS) analysis of 506 relevant genes from the liver biopsy specimen in Case 1.

Gene	Mutation	Mutant	Allelic Frequency (%)	Clinical Significance
*CHD8*	p.W1897 Exon 34 Nonsense Mutation (Suspected Germline)	c.5690G>A (p.W1897*)	41.39%	Inactivates protein, decreases β-catenin gene inhibition, causes CTNNB1 accumulation, activates Wnt pathway
***NAB2***	**NAB2-STAT6 Fusion**	**NAB2: exon7- STAT6: exon16**	**28.09%**	**Diagnostic marker for solitary fibrous tumor**
*ARID1A*	p.A333V Exon 1 Missense Mutation	c.998C>T (p.A333V)	2.38%	Involved in tumor initiation and progression
*MYCL*	p.G107R Exon 2 Missense Mutation	c.319G>C (p.G107R)	2.26%	Involved in tumor initiation and progression
*GRIN2A*	p.T888M Exon 14 Missense Mutation	c.2663C>T (p.T888M)	1.67%	Involved in tumor initiation and progression
*MLH1*	p.Q328K Exon 11 Missense Mutation	c.982C>A (p.Q328K)	1.03%	Involved in tumor initiation and progression

**Figure 2:**
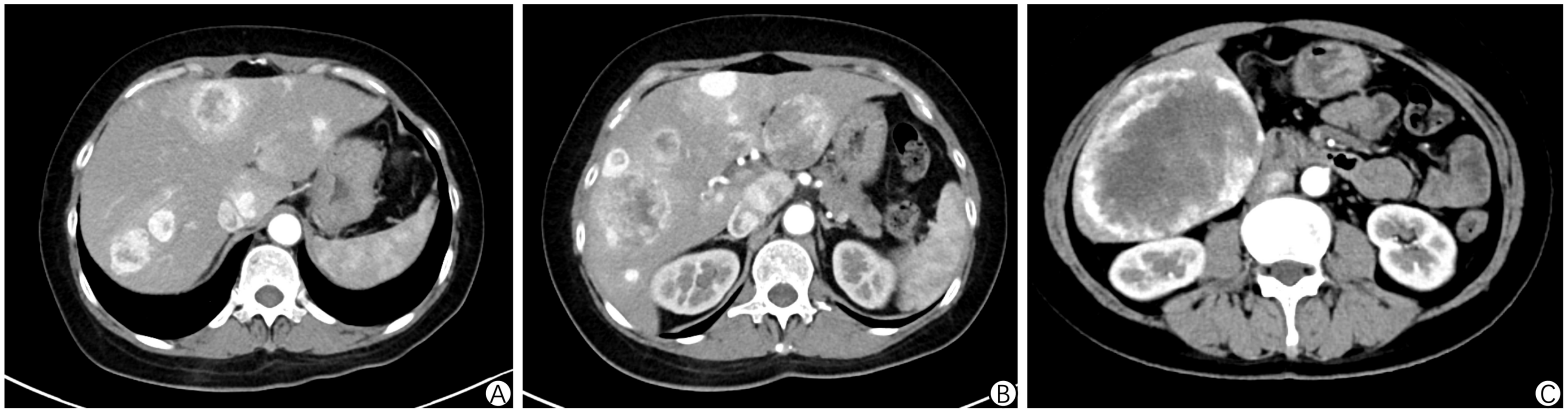
Abdominal enhanced CT in Case 1. **(A-C)** shows scattered nodular and patchy slightly low-density areas in the liver, with prominent ring-shaped enhancement. The largest lesion, located in the right posterior lobe, measures approximately 10 × 8.2 cm.

A follow-up thoracic, abdominal, and pelvic CT enhancement scan revealed scattered nodular and lamellar hypodense shadows in the liver, with marked circumferential enhancement. The largest lesion, located in the right posterior lobe, measured approximately 10 × 8.2 cm. Bone destruction was observed on the left side of the sacrum, accompanied by a lamellar soft-tissue mass measuring approximately 5.0 × 4.6 cm, showing marked inhomogeneous enhancement. A ground-glass nodule was detected in the lower lobe of the left lung, with the nature yet to be determined. Scattered small solid nodules in both lungs were considered benign and old lesions. Refer to **Figure 2**. An MR enhancement scan of the pelvis revealed bone destruction on the left side of the sacrum, with an associated soft tissue mass measuring approximately 5.8 × 3.8 × 5.5 cm. The lesion exhibited uneven signals, with high signal intensity on DWI and marked uneven enhancement on the enhancement scan. The lesion involved the adjacent left pyriform muscle, with increased signal intensity on fat-suppressed images. Bone destruction was also noted in the adjacent left iliac bone, with localized patches of high signal on fat-suppressed images.

After a multidisciplinary team (MDT) discussion, it was concluded that the tumor might originate from the sacrum, and a further puncture biopsy was recommended to determine the pathological nature. The patient subsequently underwent a puncture biopsy of the sacral lesion, with pathological results revealing ovoid, short spindle-shaped tumor cells exhibiting mild to moderate heterogeneity. These cells were arranged in bundles and grew diffusely, with visible dilated thin-walled small blood vessels. The histomorphology was consistent with SFT. Immunohistochemistry results were as follows: CK (-), EMA (-), CD34 (focal +), STAT6 (+), CD117 (-), DOG-1 (-), PDGFR (-), Desmin (-), SOX10 (-), Vimentin (+), INI1 (+), S-100 (focal +), PR (-), CD31 (-), ERG (-), TLE1 (focal +), SDHB (+), p16 (focal +), MDM2 (focal +), CDK4 (-), and Ki-67 (approximately 15%+).

**Figure 3:**
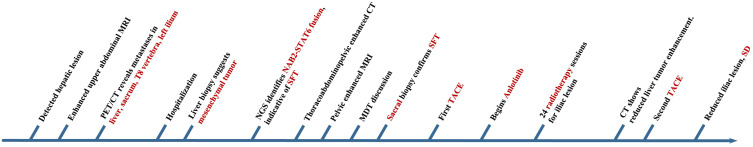
Timeline of the patient’s medical history in Case 1.

The patient underwent the first hepatic artery embolization, which involved superselective cannulation of the right hepatic artery and transcatheter injection of super-liquefied iodized oil and Embosphere microsphere particles (100-300 µm) for arterial embolization. Postoperatively, the patient started on anlotinib targeted therapy and received a total of 24 sessions of radiotherapy to the iliac lesion during the treatment period. During this period, the patient experienced lumbosacral pain, soreness, and abdominal distension, but no other significant abnormalities. Follow-up imaging after treatment showed a decrease in liver tumor enhancement. The patient then underwent a second hepatic artery embolization, with superselective cannulation of the left and right hepatic arteries for continued arterial embolization therapy. Follow-up imaging revealed shrinkage of the iliac lesion, which was evaluated as stable disease (SD). Refer to **Figure 3**.

### 2.2 Case 2

A 46-year-old male patient had a history of metastatic liver tumors for over two years. The patient had previously undergone surgery for an intracranial tumor with titanium plate implantation. Postoperative pathology suggested hemangiopericytoma, followed by local radiotherapy to the brain. Subsequent imaging revealed lesions in the pelvis, lungs, and liver, leading to pelvic tumor resection. Postoperative pathology and genetic testing confirmed SFT. The patient received long-term anlotinib treatment, along with CyberKnife therapy for liver, lung, and pelvic lesions. The patient had chronic hepatitis B, controlled and stabilized with oral entecavir. The patient had no family history of genetic diseases or tumors. Physical examination revealed stable vital signs, normal cardiopulmonary function, a soft abdomen without tenderness or rebound pain, and no palpable liver or spleen. Laboratory tests showed normal blood, urine, and fecal results, normal biochemistry, and negative tumor markers.

**Figure 4:**
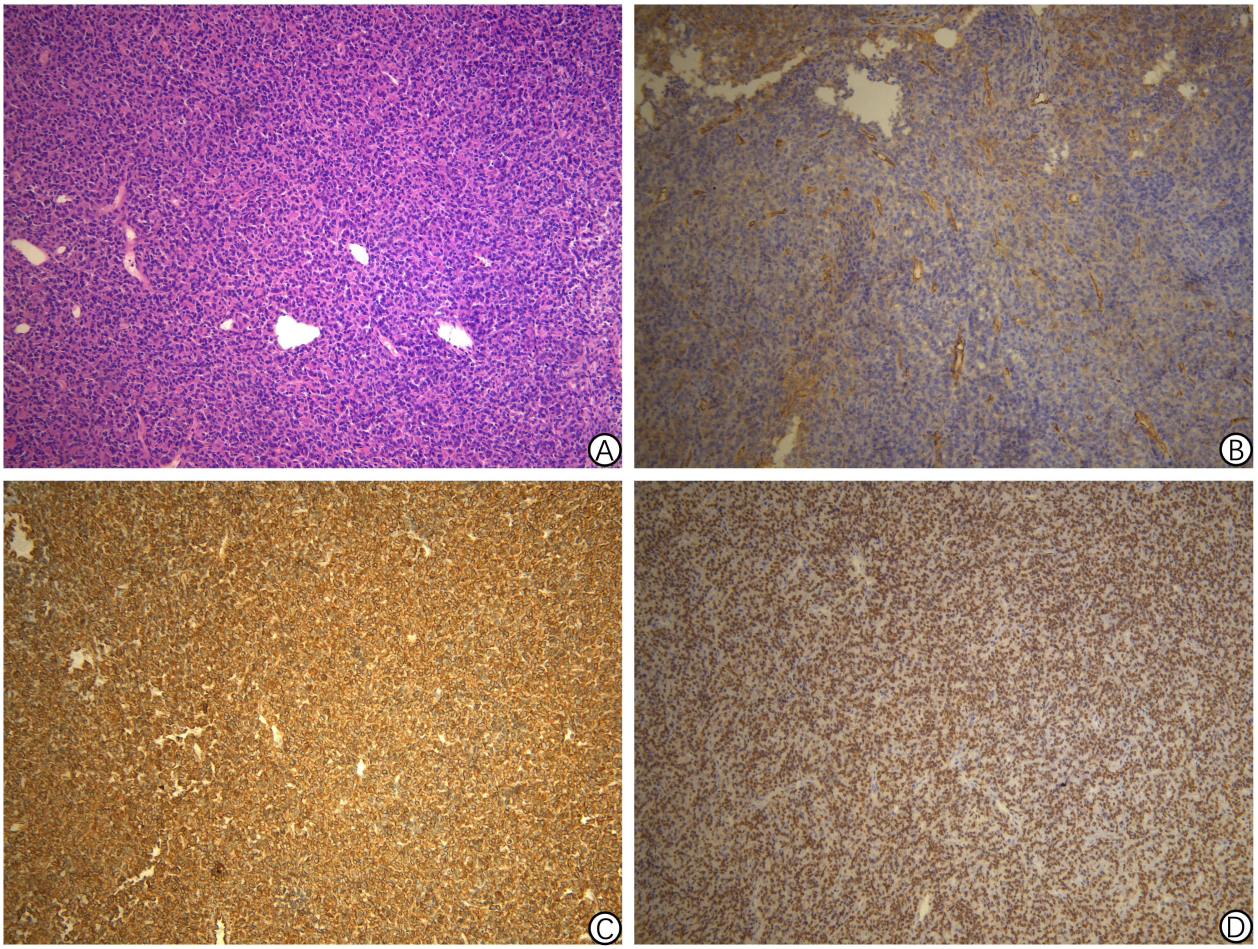
Pathology findings of solitary fibrous tumor of pelvic tumor resection specimen in Case 2. **(A)** Proliferation of spindle cells randomly arranged in the abundant stromal collagen (hematoxylin and eosin staining, 200 × magnification); **(B)** Immunohistochemical staining revealing the positive CD34 staining in the tumor cells (200 × magnification); **(C)** Immunohistochemical staining revealing the positive CD99 staining in the tumor cells (200 × magnification); **(D)** Immunohistochemical staining showing a strong STAT6 expression in the nucleus (200 × magnification).

**Figure 5:**
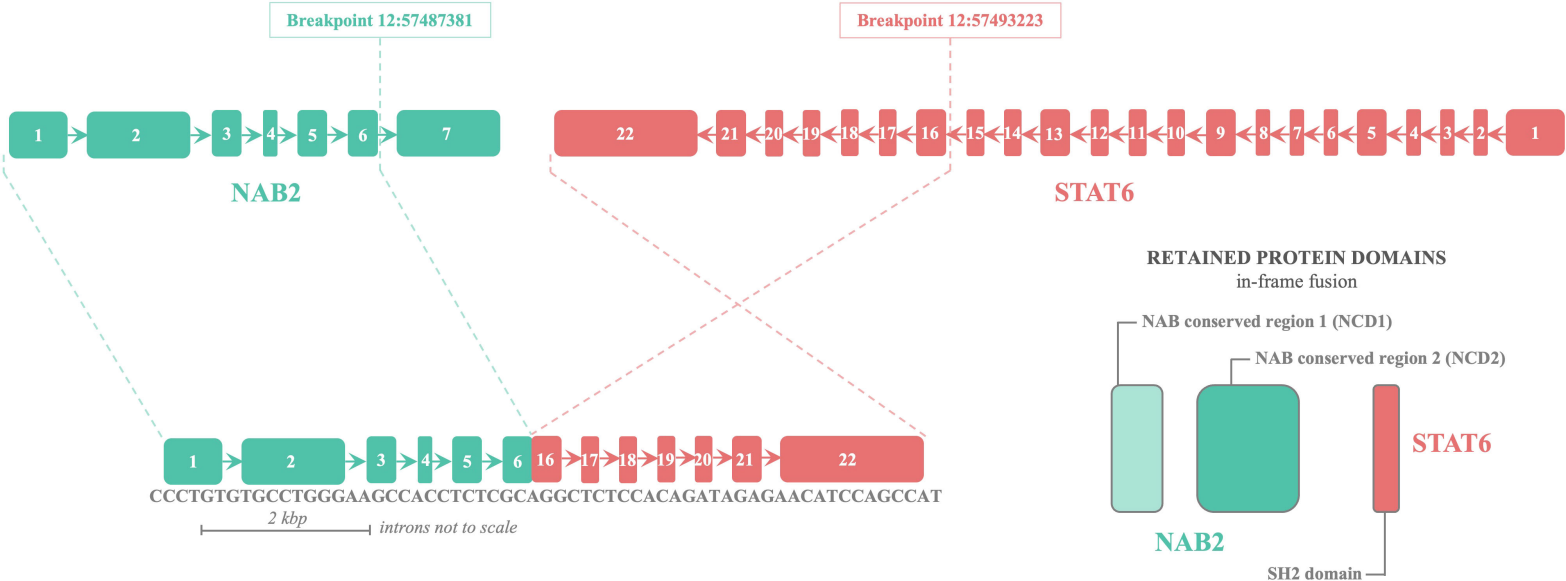
Schematic representation of NAB2–STAT6 fusion in Case 2.

Pathological examination of the pelvic tumor resection specimen revealed infiltrative growth of tumor tissue into the bone and surrounding soft tissues. The tumor consisted of ovoid and short spindle-shaped cells with heterogeneous nuclei and nuclear schizogony, arranged in bundles. Features included vitreous degeneration, ossification, and antler-like branching blood vessels, with no evidence of necrosis. Immunohistochemistry results were as follows: CK (-), EMA (-), Vimentin (+), CD99 (+), NKX2.2 (-), BCOR (partially +), WT1 (-), Ki-67 (15% +), CD34 (focal weak +), STAT6 (+), SMA (-), S-100 (-), Desmin (-), TLE1 (-), SATB2 (-), and WT1 (-).Comparing the current specimen with the patient’s previous intracranial tumor pathology slides revealed similar morphology, suggesting recurrent intracranial SFT metastasis. Refer to **Figure 4**. Subsequent genetic testing identified a NAB2-STAT6 fusion, confirming the diagnosis of SFT. Refer to **Figure 5** and **Table 2**.

**Table 2:** Comparison of genetic test results between two cases.

	Case 1	Case 2
Sampling site	Liver	Pelvis
Specimen type	Percutaneous biopsy specimen	Surgical resection specimen
Detection method	NGS	NGS
Detection range	Exons of relevant genes, introns associated with fusion, regions of alternative splicing, and specific MS loci	As left
Number of genes	506	555
Type of variation	NAB2-STAT6 Fusion	NAB2-STAT6 Fusion
Mutation region	NAB2: exon7- STAT6: exon16	NAB2: exon6- STAT6: exon16
Other Variants	Mutations in other genes such as CHD8, ARID1A, etc	No other special gene mutations

NGS, next-generation sequencing; MS, microsatellite.

**Figure 6:**
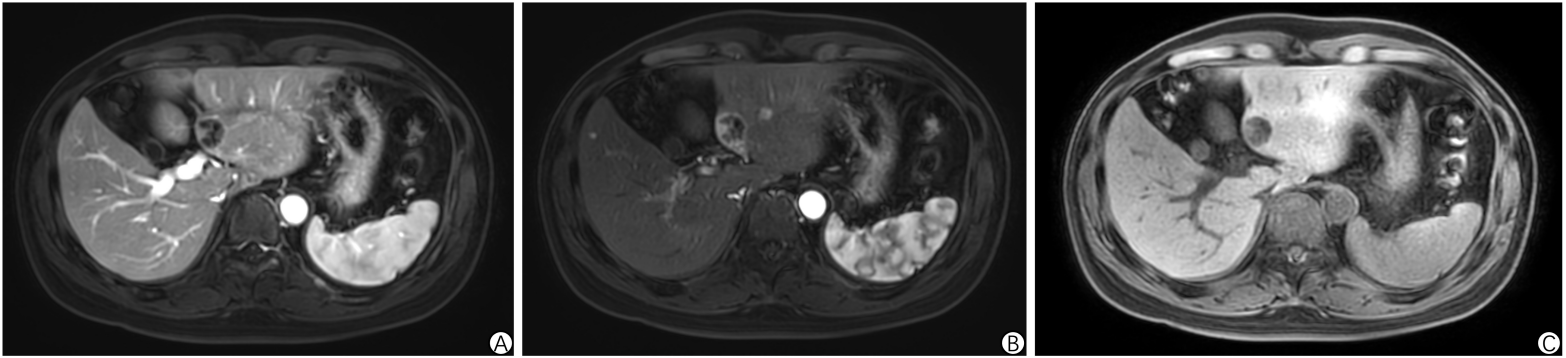
Abdominal enhanced MRI in Case 2. **(A-C)** demonstrates scattered round nodules in the liver with prolonged T1 and T2 signals. The largest nodule, located in the left lobe, measures approximately 2.5 × 2.1 cm and shows a pronounced high signal on DWI. Most nodules exhibit ring-shaped enhancement, with some displaying significant enhancement during the arterial phase and generally isointense enhancement in the portal phase.

Prior to hospital admission, an MRI enhancement scan of the upper abdomen revealed scattered round nodules in the liver with long T1 and T2 signals. The largest nodule, located in the left lobe, measured approximately 2.5×2.1 cm and showed a high signal on DWI. Most enhancement scans showed ring enhancement, while some arterial phases displayed significant enhancement in the form of small nodules, and portal phases exhibited isointense enhancement. Multiple liver metastases were considered, with the S7 segment lesion appearing smaller than before, showing a slight increase in enhancement. Other lesions were noted to have enlarged compared to previous scans. Refer to **Figure 6**. After admission, a CT enhancement scan of the cervicothoracic, abdominal, and pelvic regions showed rounded non-enhancing low-density shadows in the liver’s S6 and S4b segments, along with scattered rounded low-enhancing density shadows in the S5 and S6 segments, the largest measuring approximately 1.4×1.0 cm. A lesion in the dorsal segment of the lower lobe of the left lung was noted, raising suspicion of tumor metastasis, and sacral metastasis was considered at the S2-3 level.

**Figure 7:**
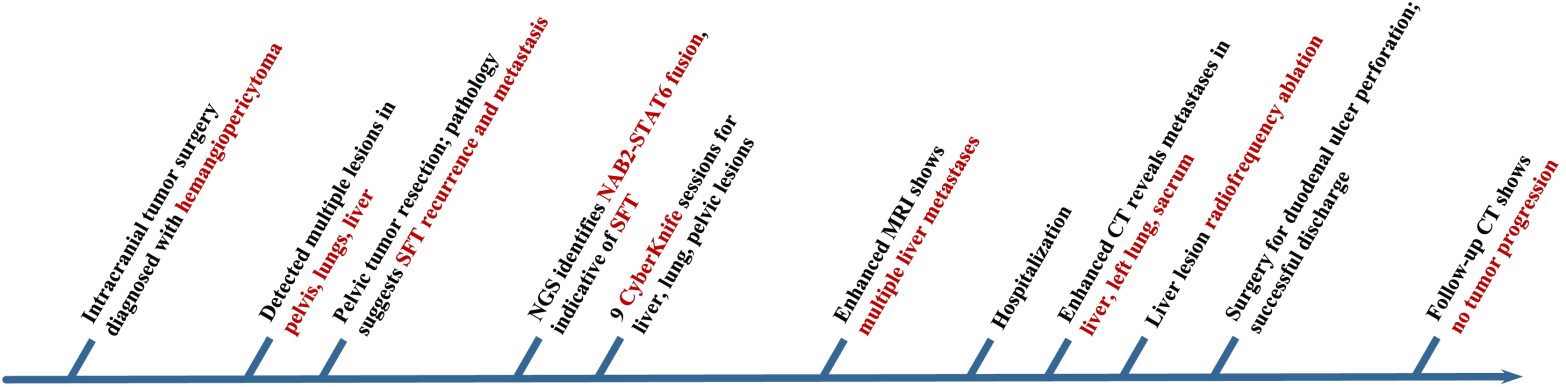
Timeline of the patient’s medical history in Case 2.

The patient was admitted to the hospital and underwent radiofrequency ablation of hepatic lesions, targeting a total of 5 tumors in both the right and left lobes of the liver. Two days after the procedure, the patient developed persistent epigastric pain. CT indicated free pneumoperitoneum, raising the suspicion of gastrointestinal perforation. The patient subsequently underwent repair of a duodenal bulb ulcer perforation. Postoperative recovery was uneventful, and regular follow-up was conducted after discharge. Repeat enhanced CT of the chest, abdomen, and pelvis showed no evidence of tumor progression. Refer to **Figure 7**.

## 3 Discussion

SFT is a rare tumor originating from mesenchymal tissue, commonly found in the chest. A clear familial inheritance pattern or association with syndromes has not been widely reported, and most SFTs are considered sporadic (1, 7). SFTL, an extremely rare subtype of SFT, affects both the young and elderly with minimal gender incidence disparity. SFTL diagnosis is challenging due to its typically asymptomatic nature, manifesting only as an abdominal mass or mild discomfort. Since 1958, only 117 cases of SFTL have been reported in the international literature, with 92 cases showing intrahepatic solitary metastases (**Table 3**). Systemic multiple metastases were observed in 27 cases, including the 2 cases reported in this study (**Table 4**).

**Table 3:** 92 cases of solitary fibrous tumor of the liver (SFTL) found in the literature.

No.	Age	Gender	Lobe	Size (cm)	Symptom	Treatment	Follow-up	Outcome	IHC					Author	Year
									Vimentin	CD34	Bcl-2	CD99	STAT6		
1	16	F	R	23	N/A	Resection	24 months	Not reported	N/A	N/A	N/A	N/A	N/A	Edmondson et al. (8)	1958
2	N/A	N/A	R	5	N/A	Resection	N/A	Not reported	N/A	N/A	N/A	N/A	N/A		
3	56	M	R	15	N/A	Radiation	2 days	Death	N/A	N/A	N/A	N/A	N/A	Nevius and Friedman (9)	1959
4	62	M	L	24	N/A	Resection	N/A	Not reported	N/A	N/A	N/A	N/A	N/A	Ishak et al. (10)	1976
5	62	F	L	23	N/A	Resection	Intraoperative death	Death	N/A	N/A	N/A	N/A	N/A		
6	27	F	L	27	N/A	Resection	6 months	Not reported	N/A	N/A	N/A	N/A	N/A	Kim and Damjanov (11)	1983
7	84	F	L	15	N/A	Resection	29 months	Disease-free	(+)	N/A	N/A	N/A	N/A	Kottke-Marchant et al. (12)	1989
8	39	F	L	18	N/A	Resection	53 months	Not reported	N/A	N/A	N/A	N/A	N/A	Kasano et al. (13)	1991
9	50	M	R	17	Abdominal pain	Resection	41 months	Disease-free	(+)	(+)	N/A	N/A	N/A	Barnoud et al. (14)	1996
10	57	M	L	18	N/A	Resection	38 months	Not reported	(+)	(+)	N/A	N/A	N/A	Levine et al. (15)	1997
11	61	F	R	20	Abdominal pain	Resection	72 months	Disease-free	(+)	(+)	N/A	N/A	N/A	Guglielmi et al. (16)	1998
12	69	F	L	N/A	N/A	Resection	12 months	Not reported	(+)	(+)	N/A	N/A	N/A	Lecesne et al. (17)	1998
13	49	M	L	17	N/A	Resection	15 months	Not reported	(+)	(+)	N/A	N/A	N/A	Bejarano et al. (18)	1998
14	62	F	N/A	23	Incidental	Resection	N/A	Not reported	(+)	(+)	N/A	N/A	N/A	Moran et al. (19)	1998
15	34	F	N/A	2	Incidental	Resection	Incidental (autopsy)	Death	N/A	N/A	N/A	N/A	N/A		
16	57	F	N/A	24	Incidental	Resection	N/A	Not reported	(+)	(+)	N/A	N/A	N/A		
17	32	M	N/A	12	Periumbilical pain	Resection	N/A	Not reported	(+)	(+)	N/A	N/A	N/A		
18	68	F	N/A	12	Incidental	Resection	Died day 2 postop	Death	(+)	(+)	N/A	N/A	N/A		
19	83	F	R	18	Fatigue/Weight lost/Hypoglycemia/Alkaline phosphatase↑	Resection	Died day 6 postop	Death	(+)	(+)	N/A	N/A	N/A		
20	72	F	L	9	Incidental	Resection	12 months	Disease-free	(+)	(+)	N/A	N/A	N/A		
21	62	M	L	24	Hematuria	Resection	N/A	Not reported	(+)	(+)	N/A	N/A	N/A		
22	50	F	N/A	3	Incidental	Resection	N/A	Not reported	(+)	(+)	N/A	N/A	N/A		
23	40	F	R	14-17	Abdominal distention/Vague right upper quadrant pain	Resection	N/A	Not reported	(+)	(+)	(+)	N/A	N/A	Fuksbrumer et al. (20)	2000
24	71	F	R	14-17	Abdominal distention/Vague right upper quadrant pain	Resection	N/A	Not reported	(+)	(+)	(+)	N/A	N/A		
25	80	M	R	14-17	Abdominal distention/Vague right upper quadrant pain	No	N/A	Not reported	(+)	(+)	(+)	N/A	N/A		
26	25	F	N/A	32	N/A	Resection	6 months	Not reported	(+)	N/A	N/A	N/A	N/A	Yilmaz et al. (21)	2000
27	75	M	N/A	21	Abdominal fullness/Weight loss/Chills & Fever	Resection	11 months	Disease-free	N/A	(+)	N/A	N/A	N/A	Lin et al. (22)	2001
28	N/A	N/A	N/A	N/A	N/A	N/A	N/A	Not reported	N/A	N/A	N/A	N/A	N/A	Gold et al. (23)	2002
29	N/A	N/A	N/A	N/A	N/A	N/A	N/A	Not reported	N/A	N/A	N/A	N/A	N/A		
30	63	F	R	30	Abdominal fullness/Weight increase/Shortness of breath/Ankle edema	Resection	6 months	Disease-free	(+)	(+)	N/A	N/A	N/A	Neeff et al. (24)	2004
31	76	F	R	20	Drenching sweat	Resection	11 months	Not reported	N/A	(+)	(+)	N/A	N/A	Chithriki et al. (25)	2004
32	65	M	R	30	Abdominal pain	Resection	30 months	Disease-free	(+)	(+)	N/A	N/A	N/A	Vennarecci et al. (26)	2005
33	73	F	R	35	Hypoglycemic	Resection	N/A	Not reported	(+)	(+)	(+)	N/A	N/A	Moser et al. (27)	2005
34	42	F	R	6	Incidental	Resection	N/A	Disease-free	N/A	(+)	N/A	N/A	N/A	Ji et al. (28)	2006
35	63	F	R	N/A	Abdominal pain	Resection	96 months	Disease-free	N/A	(+)	N/A	N/A	N/A	Lehmann et al. (29)	2006
36	61	F	R	30	Abdominal pain/Cramps	Resection	10 months	Disease-free	(+)	(+)	N/A	N/A	N/A	Nath et al. (30)	2006
37	74	M	L	24	Gastric fullness/Post-prandial nausea	Resection	12 months	Disease-free	(+)	(+)	(+)	(+)	N/A	Terkivatan et al. (31)	2006
38	70	M	R	27	Hypoglycemia	Resection	9 months	Recurrence & Metastasis	(+)	(+)	(+)	(+)	N/A	Chan et al. (32)	2007
39	52	M	L	12	Incidental	Resection	22 months	Disease-free	(+)	(+)	N/A	N/A	N/A	Obuz et al. (33)	2007
40	40	F	L	N/A	Abdominal pain	Resection	49 months	Disease-free	(+)	(+)	N/A	N/A	N/A	Perini et al. (34)	2007
41	N/A	N/A	N/A	N/A	N/A	Resection	N/A	Not reported	N/A	N/A	N/A	N/A	N/A	Weitz et al. (35)	2007
42	N/A	N/A	N/A	N/A	N/A	No	N/A	Not reported	N/A	N/A	N/A	N/A	N/A		
43	N/A	N/A	N/A	N/A	N/A	No	N/A	Not reported	N/A	N/A	N/A	N/A	N/A		
44	45	F	R	N/A	Epigastric pain	No	N/A	Not reported	N/A	(+)	N/A	N/A	N/A	Kandpal et al. (36)	2008
45	68	M	R	N/A	Hypoglycaemic	Resection	25 months	Disease-free	(+)	N/A	N/A	N/A	N/A	Fama et al. (37)	2008
46	82	F	L	18	Abdominal pain	Resection	21 months	Disease-free	(+)	(+)	(+)	N/A	N/A	Korkolis et al. (38)	2008
47	71	M	R	8.7	N/A	Resection	9 months	Not reported	N/A	(+)	(+)	(+)	N/A	Chen et al. (39)	2008
48	68	F	L+R	15	Pain of left groin	TACE	N/A	Not reported	(+)	(+)	N/A	N/A	N/A	El-Khouli et al. (40)	2008
49	30	F	R	6.7	Incidental	No	6 months	Not reported	N/A	(+)	(+)	N/A	N/A	Hoshino et al. (41)	2009
50	34	F	R	25	Abdominal pain/Abdominal distention	Resection	24 months	Disease-free	(+)	(+)	N/A	N/A	N/A	Novais et al. (42)	2010
51	54	M	R	17	Right hypochondrium pain/Weight loss	Resection	72 months	Death	(+)	(+)	N/A	N/A	N/A	Brochard et al. (43)	2010
52	62	M	L	N/A	N/A	Resection	N/A	Not reported	N/A	(+)	N/A	N/A	N/A	Haddad et al. (44)	2010
53	45	F	R	7.4	N/A	Resection	N/A	Not reported	(+)	(+)	(+)	N/A	N/A		
54	51	F	L	N/A	Incidental	Resection	N/A	Not reported	N/A	N/A	N/A	N/A	N/A	Park et al. (45)	2011
55	24	F	R	30	Abdominal pain/Abdominal distention/Vague right quadrant pain	TACE + Resection + PEIs + Chemo	16 months	Death	(+)	(+)	(+)	N/A	N/A	Peng et al. (46)	2011
56	59	M	L	9	Fatigue	Resection	24 months	Disease-free	(+)	(+)	(+)	(+)	N/A	Sun et al. (47)	2011
57	34	F	L	14.5	Dyspepsia	Resection	48 months	Disease-free	(+)	(+)	(+)	N/A	N/A	Patra et al. (48)	2012
58	85	F	L	N/A	Drenching sweats	Resection	N/A	Disease-free	N/A	(+)	(+)	N/A	N/A	Radunz et al. (49)	2012
59	66	F	R	N/A	Abdominal girth	Resection	30 months	Disease-free	N/A	(+)	N/A	N/A	N/A	Belga et al. (50)	2012
60	23	F	R	27	Nausea/Vomiting/Abdominal pain	Resection	10 months	Disease-free	(+)	(+)	(+)	N/A	N/A	Morris et al. (51)	2012
61	46	M	R	21	Abdominal pain	HRT + Chemo + Resection	10 months	Disease-free	N/A	(+)	N/A	N/A	N/A	Beyer et al. (52)	2012
62	64	M	L	N/A	Abdominal pain/Fatigue	Resection	N/A	Not reported	N/A	(+)	(+)	N/A	N/A	Soussan et al. (53)	2013
63	42	M	L	1.5	Abdominal pain	Resection	N/A	Not reported	N/A	(+)	(+)	N/A	N/A	Liu et al. (54)	2013
64	62	F	L	N/A	Abdominal pain/Weight loss	Resection	N/A	Not reported	N/A	(+)	(+)	(+)	N/A	Jakob et al. (55)	2013
65	65	M	L	N/A	Incidental	Resection	12 months	Disease-free	N/A	(+)	(+)	(+)	N/A	Debs et al. (56)	2013
66	87	F	R	14.6	Disturbances of liver function	No	10 months	Remain	N/A	N/A	N/A	N/A	N/A		
67	38	F	L	8	N/A	Resection	N/A	N/A	N/A	(+)	N/A	(+)	N/A	Durak et al. (57)	2013
68	78	M	L	17	N/A	Resection	N/A	N/A	(+)	(+)	(+)	(+)	N/A	Vythianathan and Yong (58)	2013
69	49	M	L+R	7.6	Abdominal pain	Resection	3 months	Not reported	(+)	(+)	(+)	N/A	N/A	Song et al. (59)	2014
70	68	F	L	7.5	N/A	Resection	28 months	N/A	(+)	(+)	N/A	N/A	N/A	Teixeira Jr et al. (60)	2014
71	55	F	L	17	Abdominal pain/Weight loss/Fatigue	Resection	60 months	Recurrence	N/A	(+)	(+)	N/A	N/A	Du et al. (61)	2015
72	58	M	L	15	N/A	Resection	36 months	N/A	(+)	(+)	N/A	N/A	N/A	Beltran et al. (62)	2015
73	79	F	R	15	Abdominal pain	TACE + Resection	31 months	Disease-free	(+)	(+)	(+)	N/A	N/A	Bejarano et al. (63)	2015
74	51	M	R	2.3	Epigastric fullness	Resection	11 months	Disease-free	N/A	(+)	(+)	N/A	N/A	Feng et al. (64)	2015
75	49	M	L	8.7	Fever	Resection	17 months	Disease-free	(+)	(+)	(+)	N/A	N/A		
76	51	F	R	8.4	Incidental	Resection + Adjuvant chemo	31 months	Disease-free	(+)	(+)	(+)	N/A	N/A		
77	52	F	R	12	Incidental	Resection + MWA	37 months	Recurrence	(+)	(+)	N/A	N/A	N/A		
78	65	M	L	18	Incidental	Resection	16 months	Disease-free	N/A	(+)	(+)	(+)	N/A	Silvanto et al. (65)	2015
79	40	M	L	4.7	Chest pain	Resection	N/A	Not reported	(+)	(+)	(+)	(+)	N/A	Kueht et al. (66)	2015
80	74	F	R	24	Abdominal pain	Resection	15 months	Death	(+)	(+)	(+)	N/A	(+)	Maccio et al. (67)	2015
81	80	F	R	19	Dyspnea/Cough/Asthenia/Abdominal pain	Chemo	4 months	Death	(+)	(+)	(+)	N/A	(+)		
82	65	M	R	3	Abdominal pain/Vomiting	Chemo	5 months	Death	(+)	(+)	(+)	N/A	(+)		
83	55	M	R	8.6	Annually followed up	Resection	11 months	Disease-free	N/A	(+)	(+)	(+)	N/A	Makino et al. (68)	2015
84	61	M	R	15	Loose bowel motion/Black stool	Resection	74 months	Recurrence & Metastasis	N/A	(+)	(+)	(+)	N/A	Nelson Chen and Kellee Slater (2)	2017
85	57	M	L	18	Abdominal pain	Resection	3 months	Disease-free	N/A	(+)	(+)	N/A	N/A	Shinde et al. (69)	2018
86	61	M	R	16	Hypoglycemia	TACE + Resection	5 months	Disease-free	N/A	(+)	(+)	(+)	N/A	Santos-Aguilar et al. (70)	2019
87	17	F	L	21	Abdominal pain	Resection	3 months	Disease-free	N/A	N/A	(+)	(+)	N/A	Shu et al. (71)	2019
88	32	F	R	19.5	Incidental	Autotransplantation	3 months	Disease-free	N/A	(+)	N/A	N/A	N/A	Sun et al. (72)	2019
89	54	M	R	6	Incidental	Resection	Till now	Recurrence & Metastasis	(+)	(+)	(+)	(+)	N/A	Wang et al. (73)	2021
90	59	F	R	14	Abdominal distention	TACE + Resection	12 months	Metastasis	(+)	(+)	(+)	(+)	(+)	Lin et al. (74)	2022
91	42	M	R	2.7	Abdominal pain	Resection	6 months	Metastasis	N/A	(+)	N/A	N/A	(+)	Xie et al. (75)	2022
92	49	M	L	16.5	Incidental	Resection	6 months	Metastasis	N/A	(+)	N/A	N/A	(+)	Ye et al. (76)	2023

N/A, not available; M, male; F, female; L, left; R, right; No, not operated; TACE, transarterial chemoembolization; PEIs, percutaneous ethanol injections; Chemo, chemotherapy; HRT, hormone replacement therapy; MWA, microwave ablation; IHC, immunohistochemistry.

**Table 4:** 27 cases of solitary fibrous tumor of the liver (SFTL) with malignant features, local recurrence or metastatic disease found in the literature, including the present cases.

No.	Age	Gender	Site	Size (cm)	Symptom	Treatment	Follow-up	Outcome	IHC					Genetic testing	Author	Year
									Vimentin	CD34	Bcl-2	CD99	STAT6			
1	25	F	Liver/Bone	32	Weakness/Fatigue/Anorexia/Vomiting/Progressive jaundice	Resection + Chemo	7 months	N/A	(+)	N/A	N/A	N/A	N/A	Not reported	Yilmaz et al. (21)	2000
2	70	M	Liver/Lung	27	Hypoglycemia/Progressive jaundice	TACE + Resection	12 months	Tumor progression	(+)	(+)	(+)	(+)	N/A	Not reported	Chan et al. (32)	2007
3	54	M	Liver/Bone	17	RUQ pain/Weight loss	Resection	72 months	Death	(+)	(+)	N/A	N/A	N/A	Not reported	Brochard et al. (43)	2010
4	24	F	Liver/Bone	30	RUQ discomfort/Distention	TACE + Resection + PEIs + Chemo	16 months	Death	(+)	(+)	(+)	N/A	N/A	Not reported	Peng et al. (46)	2011
5	48	M	Kidney/Liver/Lung	28	Incidental	Resection	96 months	Tumor progression	(+)	N/A	N/A	(+)	N/A	Not reported	Sasaki et al. (77)	2013
6	74	F	Liver/Lung/Omentum/Mesemtery/Abdominal wall	24	Incidental	Resection	13 months	Death	(+)	(+)	(+)	(+)	(+)	Not reported	Maccio et al. (67)	2015
7	80	F	Liver/Lung	19	Dyspnoea/Cough/Asthenia/Abdominal pain	Palliative chemotherapy	5 months	Death	(+)	(+)	(+)	(+)	(+)	Not reported		
8	65	M	Liver/Lung	3	Abdominal discomfort/Vomiting/Pain	Chemo	5 months	Death	(+)	(+)	(+)	(+)	(+)	Not reported		
9	61	M	Liver/Pleura	15	Loose bowel motion/Black stool	Resection	74 months	Tumor progression	N/A	(+)	(+)	(+)	N/A	Not reported	Nelson Chen and Kellee Slater (2)	2017
10	39	M	Brain/Liver/Pancreatic tail	20	Hypoglycemia	Resection	6 months	Tumor progression	(+)	N/A	(+)	N/A	(+)	Not reported	Andrew J. Degnan (78)	2017
11	60	F	Meninges/Liver	3	Back pain	Resection	N/A	Not reported	N/A	(+)	(+)	N/A	N/A	Not reported	Belie Lu et al. (79)	2018
12	41	M	Pelvic/Liver	14	Abdominal discomfort	Sunitinib	2.5 months	Tumor progression	N/A	N/A	N/A	N/A	(+)	NAB2-STAT6	Chuanyong Lu et al. (80)	2018
13	74	N/A	Brain/Liver	N/A	Cognitive decline/Personality changes/Urinary incontinence/Self- reported clear rhinorrhea while eating	SBRT	18 months	Tumor progression	N/A	N/A	N/A	N/A	(+)	Not reported	Reddy et al. (81)	2019
14	49	F	Bone/Liver	13.3	Malaise/Abdominal bloating	Resection	12 months	Tumor progression	(+)	N/A	N/A	N/A	(+)	NAB2-STAT6	Yugawa et al. (5)	2019
15	48	M	Pancreas/Liver/Bone	14	Fainting/Hypoglycemia	Resection + TACE	6 months	Tumor progression	N/A	(+)	(+)	N/A	(+)	Not reported	Hao Gang et al. (82)	2020
16	39	M	Brain/Liver/Lung/Right adrenal gland/Left kidney/Mesenterium/Right pubic bones	6	Abdominal pain/Fever	No further intervention	2 weeks	Death	N/A	N/A	N/A	N/A	(+)	Not reported	Maeda et al. (83)	2021
17	53	M	Pelvic/Liver/Lung/Bone/Adrenal	N/A	Abdominal pain	No further intervention	1 months	Death	(+)	(+)	(+)	(+)	(+)	NAB2-STAT6	Nonaka et al. (84)	2021
18	47	F	Brain/Liver/Thoracic spine	4.7	Incidental	Resection	N/A	Tumor progression	N/A	(+)	(+)	N/A	(+)	NAB2-STAT6	Nirupama Singh et al. (85)	2021
19	37	F	Spleen/Liver/Pancreas	3	Abdominal pain	Resection	Till now	Tumor progression	(+)	(+)	(+)	N/A	N/A	Not reported	Wending Wang et al. (73)	2021
20	54	M	Liver/Spleen/Chest wall	6	Incidental	Resection	Till now	Tumor progression	(+)	(+)	(+)	(+)	N/A	Not reported		
21	59	F	Liver/Right thoracic cavity	14	Abdominal distention	TACE + Resection	12 months	Tumor progression	(+)	(+)	(+)	(+)	(+)	Not reported	Jiajun Lin et al. (74)	2022
22	42	M	Liver/Brain	2.7	Abdominal pain	Resection	6 months	Tumor progression	N/A	(+)	N/A	N/A	(+)	Not reported	Guang-Yuan Xie et al. (75)	2022
23	67	M	Retroperitoneal/Liver/Intestine/Mesentery/Omentum	N/A	N/A	Resection	13 months	Tumor progression	(+)	(+)	(+)	(+)	(+)	Not reported	Lei Liu et al. (86)	2023
24	49	M	Liver/Pelvic/Abdomine	16.5	Incidental	Resection	6 months	Tumor progression	N/A	(+)	N/A	N/A	(+)	Not reported	Xiwen Ye et al. (76)	2023
25	59	M	Urinary bladder/Liver/Lung/Abdominal cavity	6.5	Painless hematuria	Targeted therapy	132 months	Tumor progression	N/A	N/A	N/A	N/A	(+)	NAB2-STAT6	Zengin et al. (87)	2023
**26**	**52**	**F**	**Liver/Brain/pelvis**	**10.4**	**Abdominal discomfort**	**TACE + Targeted therapy+ Radiotherapy**	**Till now**	**Stable disease**	**(+)**	**(+)**	**N/A**	**N/A**	**(+)**	**NAB2-STAT6**	**Present Case 1**	**2023**
**27**	**46**	**M**	**Liver/Lung/Brain/pelvis**	**2.5**	**Abdominal discomfort/Pain**	**CyberKnife + RFA**	**Till now**	**Stable disease**	**(+)**	**(+)**	**N/A**	**(+)**	**(+)**	**NAB2-STAT6**	**Present Case 2**	**2023**

M, male; F, female; N/A, not available; RUQ, right upper quadrant; Chemo, chemotherapy; TACE, transarterial chemoembolization; PEIs, percutaneous ethanol injections; SBRT, stereotactic radiation therapy; RFA, Radiofrequency ablation; IHC, immunohistochemistry.

Literature review and this study’s cases reveal a median patient age of 57 years (range 16-87 years). The gender distribution was balanced (53 males, 59 females, 7 unknown). Single liver tumors were distributed evenly, with 34 in the left liver, 43 in the right, 2 in both, and 13 unknown. SFTL with systemic metastases developed not only in the liver but also in the pelvis, meninges, spine, lung, pancreas, and kidney, with metastases in the bone (10 cases), lung (8), brain (7), and other sites (13). The median tumor diameter was 16.0 cm (range 1.5-35 cm). Refer to **Table 5**.

**Table 5:** Clinical summary of 119 cases of solitary fibrous tumor of the liver (SFTL).

Characteristic	Value
Age (years)	57 (16-87) *
Gender (M/F/NA)	53/59/7
Main location of single tumor (left lobe/right lobe/left and right lobe/NA)	34/43/2/13
Other metastatic sites of multiple tumors (bone/lung/brain/other sites)	10/8/7/13
Tumor diameter (cm)	16(1.5-35) *
Symptom (symptomatic/asymptomatic/NA)	72/22/25
Treatment (resection/TACE/chemotherapy/other treatments/untreated/NA)	97/10/9/11/8/2
Follow-up period after treatment (months)	12(1-132) *
Outcome (disease-free/stable disease/tumor progression/death/NA)	33/3/24/17/42
Positive IHC results (Vimentin/CD34/Bcl-2/CD99/STAT6/all above)	67/92/54/27/24/7
IHC positive rate (%) (Vimentin/CD34/Bcl-2/CD99/STAT6/all above)	56/77/45/23/20/6
Genetic testing (NAB2-STAT6/NA)	7/112

*Values are expressed as median (range); M, male; F, female; NA, not available; TACE, transarterial chemoembolization; IHC, immunohistochemistry.

This study’s two patients reported mild abdominal discomfort or pain. According to the literature, 72 cases presented with symptoms such as abdominal pain, distension, weight loss, malaise, and hypoglycemia, while 22 were asymptomatic, and the symptom status of 25 cases remains unknown. The nonspecific nature of SFTL’s clinical symptoms means that most cases are discovered incidentally during examinations. When symptoms do manifest, they are primarily due to the tumor’s mass effect or associated paraneoplastic syndromes. A few patients may exhibit Doege-Potter syndrome, characterized by non-islet-cell tumor hypoglycemia (NICTH), although this condition is rare (88). The association between SFT and hypoglycemia is attributed to abnormally high levels of insulin-like growth factor (IGF)-II in the tumor. This factor can bind to insulin and IGF-I receptors, mimicking endogenous insulin effects, increasing glucose uptake by tissues, and inhibiting growth hormone secretion and its hypoglycemia regulatory response, resulting in hypoglycemia and, in severe cases, epilepsy (89).

Notably, both patients in this study had a history of intracranial tumor surgery and subsequently developed systemic metastases, including to the liver, bone, and lungs. During follow-up, we reviewed the postoperative pathological findings from the intracranial tumor resections performed at an outside hospital. In Case 1, the pathology suggested atypical hemangiopericytoma. Immunohistochemistry results showed AE1/AE3 (+), CD31 (+), CD34 (+), CD68 (+), EMA (partially +), GFAP (-), Ki-67 (15% +), NF (-), S-100 (+), and Vimentin (+). In Case 2, the pathology suggested anaplastic hemangiopericytoma. Immunohistochemistry results revealed CD34 (+), S-100 (-), GFAP (-), PR (-), STAT-6 (+), SSTR-2 (-), Bcl-2 (partially +), CD99 (+), EMA (-), Vimentin (+), and Ki-67 (20%+). Reviewing the pathology of both patients, it is evident that their postoperative diagnoses initially considered hemangiopericytoma, and their immunohistochemistry profiles shared features characteristic of mesenchymal tumors. Similarly, a patient with SFTL and multiple systemic metastases, reported by Xie et al. in 2022, exhibited a similar disease progression (75). This patient had previously undergone intracranial tumor resection, with an initial pathology diagnosis of hemangiopericytoma, followed by liver metastases, and a final diagnosis of SFTL after surgical resection. Interestingly, as research progressed, hemangiopericytoma and SFT were found to significantly overlap in histologic features and molecular characteristics. Consequently, in the 2013 WHO Classification of Tumors of Soft Tissue and Bone, hemangiopericytoma is no longer classified as a separate tumor type but is grouped with SFT (90). This underscores the importance of considering similar patient histories, such as previous intracranial tumors, to enhance diagnostic sensitivity for SFTL in clinical practice.

SFTL’s imaging characteristics are non-specific; however, ultrasound, CT, and MRI remain the primary diagnostic modalities. Abdominal ultrasound may reveal a non-uniform mass with distinct borders, which may or may not include calcifications (71). CT scans commonly reveal heterogeneous lesion densities, displaying soft tissue density in solid components and hypodensity in cystic necrotic areas, with calcification and hemorrhage being infrequent. In enhanced CT, the solid tumor component exhibits uneven and marked enhancement during the arterial phase, with progressive or sustained enhancement in the venous and delayed phases. The characteristic “fast-in-slow-out” pattern in multiphase or dynamic scans is a hallmark of SFT (6). MRI reveals that SFTL generally displays low to medium signal intensity on T1-weighted images (T1WI) and heterogeneous, mixed low to high signal intensity on T2-weighted images (T2WI) (91). For Case 1 in this study, diagnosing SFTL based solely on imaging was challenging until pathological results were obtained, indicating the absence of typical imaging characteristics for SFTL. Furthermore, in cases of SFT with systemic metastases, PET-CT can identify intensely hypermetabolic malignant metastatic lesions, aiding in the detection of the tumor and its spread.

A definitive diagnosis of SFTL relies on histopathological and immunohistochemical analysis. HE staining reveals spindle or ovoid tumor cells, irregularly arranged and fasciculated, interspersed with abundant collagen fibers and staghorn blood vessels, featuring scant cytoplasm and an ovoid nucleus. SFT is classified as malignant if it is hypercellular and mitotically active (≥ 4 mitoses per 10 HPF) and exhibits cytologic atypia, tumor necrosis, or infiltrative margins (92). Immunohistochemistry frequently identifies SFT through positive staining for Vimentin, CD34, Bcl-2, and CD99, with CD34 serving as a critical marker to differentiate SFT from other spindle cell tumors. Nonetheless, a minor subset of SFT patients may exhibit negative immunohistochemical staining for CD34 (5). Moreover, recent research has established STAT6 as a potential key protein and a specific marker for SFT, playing a significant role in its immunohistochemical diagnosis (93). This study, combined with prior reports, found immunohistochemical positivity rates for Vimentin at 56% (67/119), CD34 at 77% (92/119), Bcl-2 at 45% (54/119), CD99 at 23% (27/119), and STAT6 at 20% (24/119). Only 6% (7/119) of patients were positive for all mentioned markers. These findings underscore the importance of these immunohistochemical markers in diagnosing SFTL.

With advancements in molecular testing, genetic testing has become increasingly valuable in diagnosing SFTL. Park et al.’s study demonstrated that the NAB2-STAT6 gene fusion, involving Nerve growth factor-induced gene A binding protein (NAB) 2 and signal transducer and activator of transcription (STAT) 6, offers better sensitivity and specificity for diagnosing SFT than traditional immunohistochemical markers (94). Originating from an inversion at the 12q13 locus, the NAB2-STAT6 fusion gene triggers the expression of the early growth response pathway, with its transcripts identifiable in 55-100% of SFT cases (95). In this study, combined with literature reviews, genetic testing revealed the NAB2-STAT6 gene fusion in seven SFTL patients, offering insights for molecular-level diagnosis of SFTL.

Recent advancements have been made in prediction models for the clinical diagnosis and treatment of SFT, marking a significant trend in the evolution of clinical diagnostics. Demicco et al.’s clinical prediction model integrates factors such as patient age, tumor size, mitotic activity, and extent of tumor necrosis to predict SFT metastasis risk, offering a comprehensive assessment of the tumor state to enhance diagnostic and therapeutic strategies (96). Zhang et al.’s recent comprehensive risk prediction model for SFT incorporates tumor mitotic counts, Ki-67(+) and CD163(+) cell densities, and MTOR mutations. This model aims to pinpoint therapeutic targets and risk factors, suggesting that a combination of immunotherapy and targeted therapy could benefit SFT patients. It heralds progress in refining SFT’s risk prediction models and developing therapeutic strategies (97).

Most SFTs exhibit benign biological behavior and have a favorable survival prognosis. Prognosis is influenced by treatment choices, as well as factors like tumor size, location, and histological characteristics. The primary treatment for SFTL involves comprehensive management centered around surgical resection. The 5-year survival rate after radical resection of SFT ranges from 59% to 100%, with a 10-year survival rate between 40% and 89%, and a recurrence rate of 5% to 20% (6). Additional treatment options include transarterial chemoembolization (TACE), chemotherapy, radiotherapy, and ablation therapy. Jin’s study summarized the effects of TACE in treating SFT, highlighting its efficacy as a locoregional treatment for some cases of SFTL (98). Gou et al.’s study, which included 42 SFT patients, demonstrated the positive role of postoperative radiotherapy in prolonging survival and achieving local control (99). Ablative therapy achieved good local tumor control in three patients with liver metastases of SFT, as reported by Krendl et al., and played a key role in multidisciplinary treatment strategies (100). Additionally, several clinical studies have employed multi-targeted tyrosine kinase inhibitors (e.g., sunitinib, sorafenib, pazopanib) for the treatment of invasive SFT, with favorable outcomes in some cases (1, 101). According to previous literature, this study reported 97 SFTL cases underwent surgical resection, 10 received TACE, 9 underwent chemotherapy, 11 received other treatments, 8 were untreated, and the treatment for 2 cases was unknown. Furthermore, two patients in this study, unable to undergo surgical resection for SFTL due to widespread metastases, received comprehensive treatment including TACE, radiotherapy, targeted therapy, and ablation therapy for systemic SFT, resulting in favorable outcomes and improved survival prognosis.

Given the unpredictable nature of SFT’s biological behavior, continuous long-term follow-up is crucial. This study’s median follow-up duration was 12 months, with outcomes as follows: 33 disease-free, 3 stable disease, 24 tumor progression, 17 deceased, and 42 unknown. Additionally, the study indicated median disease-free survival (DFS) and overall survival (OS) of 126.5 and 138.8 months (102), respectively, for SFT patients, with those undergoing surgical resection generally experiencing better long-term outcomes compared to those receiving alternative treatments. If complete tumor resection is unfeasible, combination therapy may extend survival. In addition, prognostic data on SFT with concomitant metastases are limited. In this study, 27 cases of SFTL with systemic metastases were summarized. Most patients experienced tumor recurrence and progression after treatment, likely due to the highly malignant nature of SFT. However, the prognosis for SFT patients with concomitant metastases is gradually improving with advancements in diagnosis and treatment, as well as increased focus on monitoring and follow-up. Lin et al. reported a case of giant SFTL with multiple metastases (74). The patient underwent TACE combined with surgical resection after MDT discussion. One year after surgery, no tumor recurrence was observed. In this study, two patients with SFTL and multiple metastases achieved stable disease (SD) status through effective treatment and regular long-term follow-up. Thus, a comprehensive approach involving surgical resection, consistent imaging, and extended follow-up is key to enhancing SFTL patients’ survival prognosis.

In summary, SFTL, a rare tumor, often presents no typical early symptoms. Larger lesions, however, can lead to abdominal pain, distension, and potentially hypoglycemia. Clinical symptoms and imaging features of SFTL are nonspecific. Histopathology and immunohistochemistry remain the diagnostic gold standards, while genetic testing offers further diagnostic clarification. Radical resection is preferred for solitary tumors. Malignant tumors with recurrence or metastasis risk typically require combination therapy following surgical resection, with regular follow-up being crucial for enhancing patient prognosis. This study’s two cases offer insights into diagnosing and treating SFTL with systemic metastases. As more cases accumulate, it is anticipated that SFTL diagnosis and treatment strategies will evolve, subsequently improving patient long-term prognosis.

## Data Availability

The original contributions presented in the study are included in the article/supplementary material. Further inquiries can be directed to the corresponding authors.
